# Vision in high-level football officials

**DOI:** 10.1371/journal.pone.0188463

**Published:** 2017-11-21

**Authors:** António Manuel Gonçalves Baptista, Pedro M. Serra, Colm McAlinden, Brendan T. Barrett

**Affiliations:** 1 Centre of Physics (Optometry), University of Minho, Campus de Gualtar, Braga, Portugal; 2 School of Health Professions, Faculty of Health and Human Sciences, Plymouth University, United Kingdom; 3 Department of Ophthalmology, Glangwili Hospital, Hywel Dda University Health Board, Carmarthen, United Kingdom; 4 School of Ophthalmology and Optometry, Wenzhou Medical University, Wenzhou, Zhejiang, China; 5 School of Optometry & Vision Science, University of Bradford, Richmond Road, Bradford, United Kingdom; University of Waterloo, CANADA

## Abstract

Officiating in football depends, at least to some extent, upon adequate visual function. However, there is no vision standard for football officiating and the nature of the relationship between officiating performance and level of vision is unknown. As a first step in characterising this relationship, we report on the clinically-measured vision and on the perceived level of vision in elite-level, Portuguese football officials. Seventy-one referees (R) and assistant referees (AR) participated in the study, representing 92% of the total population of elite level football officials in Portugal in the 2013/2014 season. Nine of the 22 Rs (40.9%) and ten of the 49 ARs (20.4%) were international-level. Information about visual history was also gathered. Perceived vision was assessed using the preference-values-assigned-to-global-visual-status (PVVS) and the Quality-of-Vision (QoV) questionnaire. Standard clinical vision measures (including visual acuity, contrast sensitivity and stereopsis) were gathered in a subset (n = 44, 62%) of the participants. Data were analysed according to the type (R/AR) and level (international/national) of official, and Bonferroni corrections were applied to reduce the risk of type I errors. Adopting criterion for statistical significance of p<0.01, PVVS scores did not differ between R and AR (p = 0.88), or between national- and international-level officials (p = 0.66). Similarly, QoV scores did not differ between R and AR in *frequency* (p = 0.50), *severity* (p = 0.71) or *bothersomeness* (p = 0.81) of symptoms, or between international-level *vs* national-level officials for *frequency* (p = 0.03) or *bothersomeness* (p = 0.07) of symptoms. However, international-level officials reported less *severe* symptoms than their national-level counterparts (p<0.01). Overall, 18.3% of officials had either never had an eye examination or if they had, it was more than 3 years previously. Regarding refractive correction, 4.2% had undergone refractive surgery and 23.9% wear contact lenses when officiating. Clinical vision measures in the football officials were similar to published normative values for young, adult populations and similar between R and AR. Clinically-measured vision did not differ according to officiating level. Visual acuity measured with and without a pinhole disc indicated that around one quarter of participants may be capable of better vision when officiating, as evidenced by better acuity (≥1 line of letters) using the pinhole. Amongst the clinical visual tests we used, we did not find evidence for above-average performance in elite-level football officials. Although the impact of uncorrected mild to moderate refractive error upon officiating performance is unknown, with a greater uptake of eye examinations, visual acuity may be improved in around a quarter of officials.

## Introduction

Football officials are crucial participants in the professional game, implementing the rules of a game where billions of euros are at stake in Europe alone [1]. They are naturally subjected to the same environmental conditions as the players, and their basic visual demands, while not the same as the players, share many similarities. In some ways, the visual demands of officials are greater than those of the players since officials are constantly required to make decisions and they only have a brief temporal window in which to make a decision. In an era where multiple, high spatial- and temporal-resolution television cameras, are placed in various locations around the ground to facilitate detailed viewing in real time as well as high-resolution, slow-motion replays, professional football officials perform under high scrutiny. The match is controlled by a referee who has full authority to enforce the Laws of the Game [2]. Based on the observation of the body language, it has been estimated that referees make about 137 decisions per game, 64% of which are based on communication with the assistant referees and/or the fourth official [3]. The assistant referee (previously known as the ‘linesman’) is usually positioned off the field of play, along the side line, and has specific tasks, including indicating to the referee when the ball is off the field of play and when attacking players are offside [4]. They also have a role in signalling to the referee when a foul has been committed in an area of the field close to where they are located.

Successful officiating demands full knowledge and correct application of the rules of the game, and it requires excellent perceptual-cognitive skills, which will involve knowing where and when to look and the successful reading of anticipatory cues [3]. Since the information needed to officiate the game role is largely visual in nature and the official will frequently be a considerable distance away from the play, one might expect basic visual abilities to play a crucial role in determining the success with which the task of officiating can be completed.

The visual demands of different sports have been empirically studied [5, 6] and there is a strong view that the demands can differ greatly from sport to sport. Note that by “vision”, we are not necessarily referring to “visual acuity” as there are many other measures of ‘vision’ which can be deployed [7]. Certainly, the ability to resolve fine details, visual acuity, particularly under dynamic conditions might be critical, since officials and players are constantly in motion. Other visual skills that may be important include an ability to quickly achieve a clear and stable view at different viewing distances by altering accommodation and vergence. Also contrast sensitivity may be important as measures reflect performance when conditions are sub-optimal, such as when there is rain or fog.

Compared to vision in sports-players, the basic visual characteristics of officials have received much less attention. In one of the very few studies of basic visual characteristics in sports officials, high-level football referees were found to have better performance than novice referees and non-athletes in some visual skills evaluated at near and intermediate distance, including accommodation facility, peripheral vision, saccadic eye movements and speed of recognition [8]. The difference between expert and novice referees suggests that these visual skills may be important for officiating in football [8]. In a subsequent study by the same research team, when expert referees were split into two groups according to their ability in on-field decision-making, the group with better scores showed superior visual skills in visual memory, visual reaction time, peripheral vision, recognition speed, saccadic eye movements, and accommodation facility [9]. Recently, it was shown that elite referees employ a more effective visual scan pattern than sub-elite level referees when assessing foul play situations [10]. Thus there is growing evidence that vision may differ in elite versus sub-elite level football officials. However, the differences which have been found to date appear to relate more to how vision is used (e.g. visual strategies about where and when to look) rather than to acuteness of vision *per se* [10]. This distinction between vision and visual strategy in sport has been likened, respectively, to the hardware versus software distinction in computing [11].

Football officials perform outdoors in variable environmental conditions, particularly in respect to weather and illumination. From the point of view of clinical assessment of vision it is difficult or impossible to reproduce in the testing room, the range of visual conditions that are typically encountered in the field. Thus, to get a more complete picture of visual abilities, it is useful to assess not only the level of vision as measured clinically, but also the quality of vision from the official’s own perspective.

This study is concerned with the question of level of vision (i.e. basic visual capabilities, ‘hardware’) exhibited by a sample of elite football officials. There is at present no vision standard for football officiating and the nature of the relationship between officiating performance and level of vision is unknown. As a first step in characterising this relationship, we report on clinically-measured vision and on the perceived level of vision in elite-level, Portuguese football officials. Specifically, our research questions were as follows: (1) what are the visual habits of elite football officials, in relation to eye examination frequency and refractive correction worn on the pitch? (2) do perceived levels of vision and (3) basic vision abilities in elite football officials differ between different roles (referees versus assistant referees) and levels of expertise (national- versus international)? (4) how do basic visual abilities and perceived levels of vision in elite football officials compare to published norms for young adults?

## Methods

### Participants

**Fig 1:**
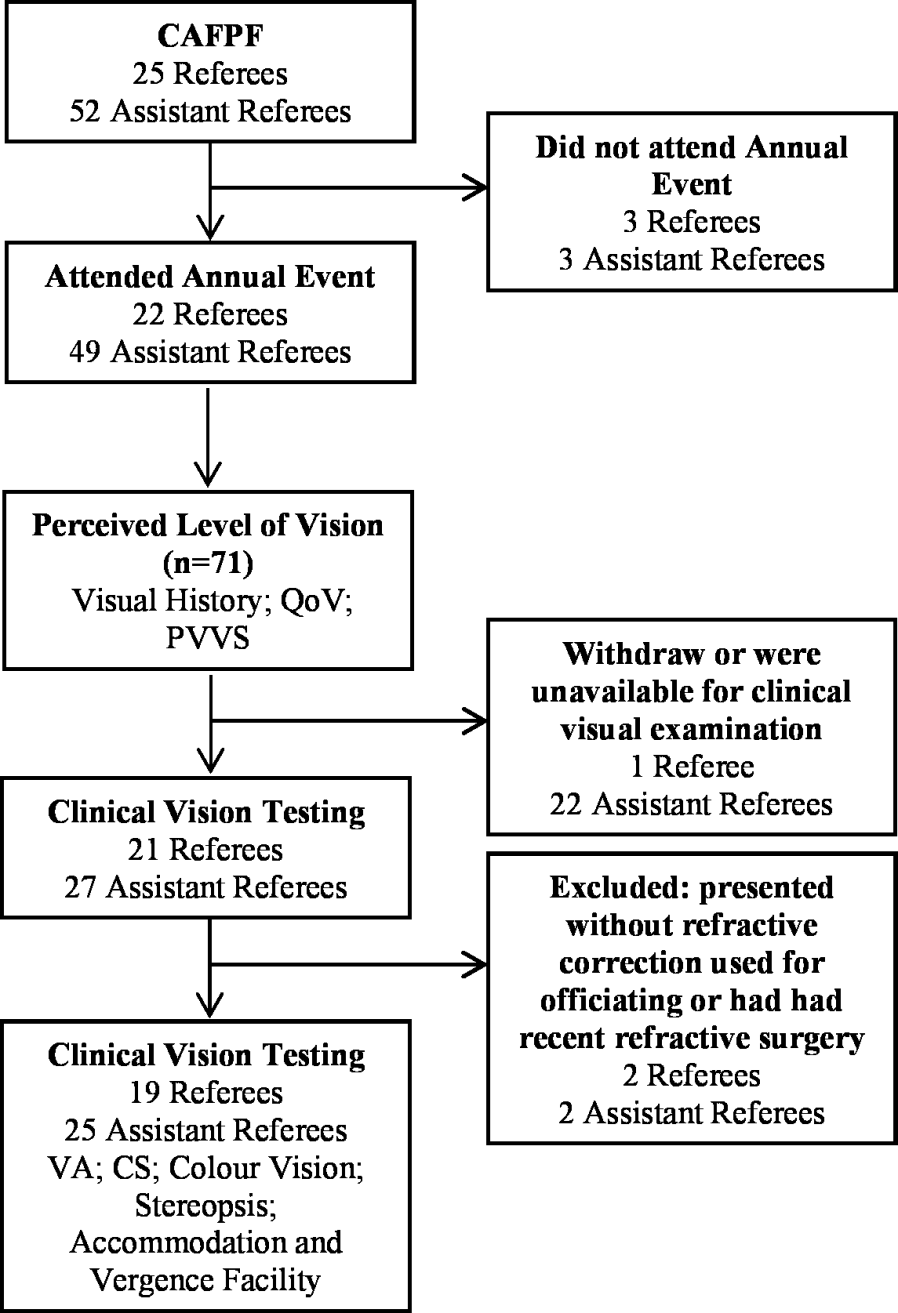
Flow chart summarizing participant recruitment. CAFPF: Conselho de Arbitragem da Federação Portuguesa de Futebol (Portuguese Referee's Committee); QoV: quality of vision questionnaire [12]; PVVS: rating-scale to measure the preference values that officials assign to their global vision status [13]; VA: visual acuity; CS: contrast sensitivity.

Data were collected in the 2013/14 season during a meeting of elite football referees (R) and assistant referees (AR) from Federação Portuguesa de Futebol (FPF). A total of 71 participants were eligible for this study (Fig 1), representing 92% of the total number of elite Portuguese football officials in that season. From the total of 25 Portuguese R (nine of whom were international-level), 22 participated in this event, including all of the internationals; three referees did not attend this meeting. The age (mean±SD) of the 22 R was 37.8±2.7 (range, 33–43 years) and all were Caucasian. From the total of 52 Portuguese AR (10 of whom were international-level), 49 participated in this event, including all internationals; again three AR did not attend this meeting. The mean age of the 49 AR was 38.1±3.6 (range, 30–44 years) and again all were Caucasian.

Every R and AR had undergone a compulsory, annual, medical exam (CAME) required by Portuguese law and conducted by a medical physician who specializes in sports medicine. This medical exam has recommended guidelines relating to the assessment of general health, also defined by Portuguese law. As part of the CAME, there is a basic assessment of vision, consisting of an evaluation of distance visual acuity (VA) with and without refractive correction, but there is no set minimum VA standard. No other tests, objective or subjective, are carried out aside from VA measurement but according to the case history physicians may refer the athlete to a vision specialist [14]. Written informed consent was obtained from all participants in this study and the research was conducted in accordance with the guidelines promoted by the Declaration of Helsinki. This study received ethical approval by the Ethics Subcommittee for Life and Health Sciences of Minho University.

### Visual history, current refractive correction worn and perceived level of vision

Visual history was obtained from all 71 participants who attended the meeting. This included the time interval since the last eye exam, excluding CAME, the type of refractive correction, if any, worn both in the daily life and when officiating on the pitch.

We used two instruments to determine how well participants rate their vision. Firstly, we used a rating-scale technique to measure the preference values assigned to global vision status (PVVS) [13]. We asked the officials to rate their current vision on a scale from 0 (completely blind) to 10 (perfect vision). In addition, we administered the Quality of Vision (QoV) questionnaire [12]. The QoV has become an important tool to measure visual symptoms [15] and uses ten visual symptoms: glare, haloes, starbursts, hazy vision, blurred vision, distortion, multiple images, fluctuation in the vision, focusing difficulties and about difficulties judging distance or depth perception. The questionnaire includes photographs to simulate the first seven visual symptoms referred to above. The questionnaire is scored on a 0–100 scale across three subscales (Frequency, Severity and Bothersome) with higher scores indicating poorer quality of vision [16, 17]. The QoV was initially developed and validated in English, but it has been administered in several, non-English speaking countries using translated versions [18–20]. A Portuguese version of the questionnaire was used in this study. When completing the PVVS and QoV, participants were not given any specific instructions as to whether they should complete this in respect of the level of vision when officiating, or for the level of vision in everyday life.

### Clinical measurement of vision

In the time available to us for clinical vision testing it was only possible to gather data from only 48 of the 71 participants who had completed the visual history and questionnaire elements of the study (Fig 1). The clinical testing was not scheduled based on visual needs but based on availability of officials’ agenda during the FPF meeting. Thus, as far as we can tell, there was no bias created by only testing a subset of eligible participants who presented for participation in a pseudo-random order. This clinical testing was performed using the same refractive correction normally used when officiating (e.g. wearing contact lenses if these were worn when on the pitch). Some of the officials we examined are very high-profile individuals who are well known to the public in Portugal. Hence we cannot say for certain the extent to which the vision testing was conducted blind to the R or AR status of the participant. However, clinical measures of vision were gathered without access to officials’ visual history or completed questionnaires (QoV and PVVS).

Clinical vision data were gathered in 21R. The results from two R were excluded because they presented without the refractive correction (contact lenses) they normally used for officiating. Thus data analysis was performed for 19R, of whom eight were international-level referees. The mean age of these participants was 37.8±2.9 (range, 33–43 years). Clinical vision data were gathered in 27AR. Again, the results for two had to be excluded because one presented without the contact lenses they normally used for officiating and the other had undergone refractive surgery (LASIK) just two weeks beforehand. Thus, analysis of the clinical visual data was performed for 25AR, of whom six were international-level. The mean age of the 25 AR participants was 38.3±3.7 (range, 31–44 years).

### Visual acuity (VA)

VA was measured at 4 meters using a logMAR chart (ETDRS: Precision Vision, CAT. NO. 2121) with a standardized, retro-Illuminated cabinet, and with a luminance of 85 cd/m^2^ (Precision Vision, SKU 2425). This chart has five letters per line ranging from 1.0 logMAR to -0.3 logMAR. A per letter scoring system was used, as advocated [21]. Visual acuity was measured, first monocularly (right eye before left eye) and then binocularly using a standard procedure [22]. Near VA was measured at 40 cm using a near logMAR visual acuity chart (GOOD-LITE #729000) with similar procedure to that used for distance VA measurement.

In order to assess potential improvements in VA associated with uncorrected refractive error, a pinhole disc (PH) was used [23]. If VA improves when viewing through a pinhole this suggests that the current refractive status is non-optimal, suggesting that better VA may be achievable with a new or updated refractive correction. Thus the pinhole is a screener for undiagnosed or altered refractive error [23]. Considering that the reliability of the ETDRS logMAR chart is ±5 letters (95% CI) [24, 25], participants who improved by 5 or more letters (i.e. one or more line(s) of letters on the ETDRS chart) with the PH were identified as likely to benefit from a new or updated optical correction.

### Contrast sensitivity (CS)

Unlike VA measures which assess the ability to read pure black letters on a white background, contrast sensitivity (CS) reflects the ability to identify targets of lower contrast (e.g. grey on a white background). There is strong evidence that CS provides a better examination of how well people ‘see’ in the real world where object contrast is seldom maximal [26]. We measured CS from a distance of 1 meter (luminance of 60 cd/m^2^) using the Pelli-Robson letter contrast sensitivity chart (Precision Vision, CAT. NO. 5014). The CS was measured monocularly and binocularly using a standard procedure [22].

### Stereoacuity

Stereoacuity was assessed using the TNO stereo test (LAMÉRIS OOTECH, 17th edition) using a standard procedure [27]. In this test, two dot patterns, one printed in red and the other in cyan (roughly complementary colours), are superimposed. A red filter is worn over one eye and a cyan filter is worn over the fellow eye. The result is that different stimuli are presented to the two eyes, and the disparity when fused simulates real depth in the two-dimensional patterns. Those with measurable stereoacuity had their result recorded using a scale which ranges from 480 to 15 seconds of arc, where lower numbers represent better performance. There are other clinical tests available to measure near stereoacuity (e.g. those featuring the use of polaroid filters, and therefore which do not create rivalrous right and left eye conditions but which, like the TNO, do not contain monocular cues). However, the TNO test is a long-established and recognised means for clinical estimation of stereoacuity [28].

### Accommodative facility

Accommodative facility refers to an individual’s ability to rapidly alter accommodation (focus) in response to a change in the accommodative demand required to keep a target clearly focussed. This was tested binocularly using ±2.00DS flippers using a standard approach [27]. One cycle consisted of the participant indicating that they had made clear, from a viewing distance of 40cm, a small body of text on the near logMAR chart following the introduction of both the +2.00DS and the -2.00DS lenses. The target was one logMAR line larger than the binocular near visual acuity measured previously. The test result is recorded as the number of +2.00/-2.00 DS cycles that the participant was able to clear in a one minute testing period.

### Vergence facility

Vergence facility is a useful means for evaluating the ability of the binocular vision to make eye movements to preserve single vision following the introduction of prism power to shift the image of the object of interest off the fovea. We tested vergence facility using a 3^Δ^ base-in (increasing divergence) and a 12^Δ^ base-out (increasing convergence) prism flippers. The task of the participant was, following the introduction of the prism power, to report as soon as the target had become single and clear again. A standard procedure was used and the procedure was similar to that used for the accommodation facility test [27]. The number of cycles (overcoming both the base-out and base-in prism power) completed in one minute was recorded as the vergence facility result.

### Colour vision

Screening for a red-green colour deficiency was conducted using the Ishihara test (Ishihara’s test chart for colour deficiency, Concise Edition, 2003; Kanehara Trading Inc., Tokyo, Japan). The test consists of 14 plates and each plate contains coloured dots of varying size. The dots form a number or pattern clearly visible to those with normal colour vision, and invisible, or which appears different to those with a red-green colour deficiency. The test was used in accordance with manufacturer’s instructions. Participants were classed respectively, as colour-normal or colour-deficient.

### Statistics

The arithmetic mean and the standard deviation (mean±SD) were used as the main measures of central location and spread, respectively, for variables with a normal distribution. For variables that were not normally distributed, or for variables with an ordinal-scale, we used the median and interquartile range (IQR). We checked for normality of the distribution for QoV parameters (frequency, severity and bothersome), distance and near VA, for CS, accommodative facility and vergence facility using the Shapiro-Wilk test. The two categories of official (R and AR) were split into subcategories of international- and national-level. The internationals in this subcategory were the national referees selected by the Portuguese Referee's Committee to be nominated by FIFA as the Portuguese International Referees for the year 2014. For normally distributed variables, a two-way factorial analysis of variance (ANOVA) was used to study the main effects of category and subcategory, and the interaction effect between category and subcategory. This applied to the QoV parameters and to the distance VA. For non-normally distributed results, we used the aligned rank transform (ART) for nonparametric factorial analyses using ANOVA procedures [29] to study the main effects of category (R & AR) and subcategory (national and international), and any interaction between category and subcategory. The ART relies on a pre-processing step that “aligns” data before applying averaged ranks, after which common ANOVA procedures can be used [29]. This procedure was applied to the remaining variables (PVVS, near VA, CS, accommodative facility, vergence facility, stereoacuity) but not colour vision for which we merely determined the proportion of each sample, and of the sample as a whole, in whom a colour-vision deficiency existed. All clinical visual test results except colour vision were correlated (using spearman-rank) with PVVS and with QoV, using data from the 44 participants in whom clinical data were gathered. The QoV and PVVS scores were also correlated with one another using the data from all 71 participants. In addition, the ratings of officials by the Portuguese Federation during season 2013/14 were correlated with our clinical test results and with our questionnaire results. Since R and AR are rated by the Federation using different criteria despite using the same rating scale, the correlations were performed separately for the two types of official. *P*-values <0.05 were deemed to indicate statistical significance but when multiple comparisons were made, Bonferroni adjustments were made to *p*-values (*e*.*g*. *p*-values were divided by the number of pair-wise tests) as a protection against Type I error. Data were analysed using the statistical package ARTool [30] and SPSS 20 (SPSS, Chicago, IL).

## Results

### Visual history and refractive correction

Refractive correction habits and visual history of the participants by category (R or AR) and subcategory (international versus national) are summarized in Table 1. Aside from CAME, the majority of participants (60.6%) had an eye exam during the previous 12 months. However, 18.3% had either never had an eye examination or if they had one, it was more than 3 years previously. The remainder (21.1%) had had an eye exam between 1 and 3 years previously.

**Table 1:** Visual history and refractive correction in referees and assistant referees by subcategory (international and national).

			Referee				Assistant Referee				All Participants n = 71 n (%)
			International n = 9 n (%)		National n = 13 n (%)		International n = 10 n (%)		National n = 39 n (%)		
**Time of last eye examination (years)**		Never	0	(0.0)	1	(7.7)	1	(10.0)	2	(5.1)	4 (5.6)
		time ≤ 1	4	(44.4)	8	(61.5)	6	(60.0)	25	(64.1)	43 (60.6)
		1 < time ≤ 2	2	(22.2)	1	(7.7)	1	(10.0)	6	(15.4)	10 (14.1)
		2 < time ≤ 3	0	(0.0)	1	(7.7)	1	(10.0)	3	(7.7)	5 (7.0)
		time > 3	3	(33.3)	2	(15.4)	1	(10.0)	3	(7.7)	9 (12.7)
		***Total***	***9***	***(100)***	***13***	***(100)***	***10***	***(100)***	***39***	***(100)***	***71 (100)***
**Refractive correction (daily life)**	No refractive surgery	No refractive correction at all	7	(77.8)	9	(69.2)	7	(70.0)	24	(61.5)	47 (66.2)
		Now has CLs & glasses	0	(0.0)	2	(15.4)	1	(10.0)	11	(28.2)	14 (19.7)
		Wears glasses only	1	(11.1)	1	(7.7)	0	(0.0)	3	(7.7)	5 (7.0)
		Wears CLs only	1	(11.1)	1	(7.7)	0	(0.0)	0	(0.0)	2 (2.8)
	Had refractive surgery	Now has no need for any refractive correction	0	(0.0)	0	(0.0)	1	(10.0)	1	(2.6)	2 (2.8)
		Now has CLs & glasses	0	(0.0)	0	(0.0)	1	(10.0)	0	(0.0)	1 (1.4)
		Now wears glasses only	0	(0.0)	0	(0.0)	0	(0.0)	0	(0.0)	0 (0.0)
		Now wears CLs only	0	(0.0)	0	(0.0)	0	(0.0)	0	(0.0)	0 (0.0)
	***Total***		***9***	***(100)***	***13***	***(100)***	***10***	***(100)***	***39***	***(100)***	***71 (100)***
**Refractive correction worn on the pitch**		Now has CLs & glasses	0	(0.0)	0	(0.0)	0	(0.0)	0	(0.0)	0 (0.0)
		Wears glasses only	0	(0.0)	0	(0.0)	0	(0.0)	0	(0.0)	0 (0.0)
		Wears CLs only	1	(11.1)	3	(23.1)	2	(20.0)	11	(28.2)	17 (23.9)

Of the 71 participants, three (4.2%) had undergone refractive surgery (LASIK) and of these, two had no residual need to wear correction whilst officiating; one of the three continued to wear contact lenses whilst on the pitch. For the remaining 68 participants (95.8%), 21 used some form of refractive correction, though the correction was not always used when officiating. For the 22 subjects who had some form of refractive correction (21 plus one who had undergone refractive surgery), 17 (23.9% of the overall sample of 71 participants) used their correction whilst officiating, and in all cases this correction consisted of contact lenses. Across the entire sample, three participants (4.2%) had undertaken vision therapy for an orthoptic problem (one for amblyopia and the other two were not able to specify), and one (1.4%) had undertaken vision therapy in an attempt to optimize vision performance.

### Perceived level of vision (PVVS)

For the purpose of Bonferroni adjustment, we considered PVVS and the three tests of QoV (see below) which resulted in a critical *p*-value of 0.013 (0.05/4). PVVS scores are provided in Table 2. Neither category (R and AR) nor subcategory (international- and national-level officials) or interaction (category versus subcategory) were statistically significant (category: F(df = 1) = 0.021, p = 0.88; sub-category: F(df = 1) = 0.197, p = 0.66; interaction: F(df = 1) = 2.338, p = 0.13).

**Table 2:** Values assigned to global vision status (PVVS) and quality of vision questionnaire (QoV).

		Referee		Assistant Referee		Statistics p_cat_; p_subcat_
		International; n = 9	National; n = 13	International; n = 10	National; n = 39	
**PVVS** **Median (Interquartile Range)**		9 (1.0)	8 (1.0)	8 (0.5)	8 (1.0)	0.88; 0.66
**QoV** **Mean±SD**	Frequency^§^	49±16	62±7	56±13	60±13	0.50; 0.03
	Severity^§^	40±18	57±14	48±12	53±15	0.71; 0.009*
	Bothersome^§^	48±27	65±19	56±16	60±20	0.81; 0.07

Data are from 71 participants.

^§^Variable with normal distribution. PVVS is represented by median and interquartile range [median (IQR)] with higher scores indicating better vision. QoV is represented by mean and standard deviation (mean ±SD) with higher mean values indicating poorer perceived quality of vision. The statistical significance of differences in the perceived level of vision between referees and assistant referees (p_cat_) and between international- and national-level officials (p_subcat_) was examined (*p<0.01); see right-most column.

### Quality of vision (QoV)

The effect of category (R versus AR) was not significant in relation to *Frequency* (F(df = 1) = 0.464, p = 0.50), *Severity* (F(df = 1) = 0.138, p = 0.71) or *Bothersome* (F(df = 1) = 0.062, p = 0.81) (Table 2). Similarly, the effect of sub-category (international versus national) was not statistically significant in relation to *Frequency* (F(df = 1) = 4.984, p = 0.03) or *Bothersome* (F(df = 1) = 3.307, p = 0.07) but was significant in relation to *Severity* (F(df = 1) = 7.340, p = 0.009) (Table 2). Specifically, international-level officials reported less severe symptoms compared to national-level officials. None of the interactions between category and sub-category were significant (*Frequency*: F(df = 1) = 1.573, p = 0.21; *Severity*: F(df = 1) = 2.208, p = 0.14; *Bothersome*: F(df = 1) = 1.317, p = 0.26).

### Clinically-measured vision

For the purposes of Bonferroni adjustment, we considered all the basic vison tests used with exception of colour vision, resulting in a critical *p*-value of 0.004 (i.e. 0.05/12). The results of the vision tests by category (R compared to AR) and subcategory (national- versus international-level) are shown in Table 3. Statistical comparison reveals that none of the vision test results differed as a function of category or subcategory (Table 3).

**Table 3:** Clinical visual measures from 44 participants.

Test		Referee		Assistant Referee		All n = 44	Stat p_cat_; p_subcat_
		International; n = 8	1^st^ Category; n = 11	International; n = 6	1^st^ Category; n = 19		
**Distance visual acuity†** **(logMAR)**	Right eye	-0.10±0.08 -0.12 (0.11)	-0.09±0.08 -0.10 (0.10)	-0.12±0.09 -0.13 (0.15)	-0.09±0.09 -0.10 (0.12)	-0.10±0.08 -0.10 (0.10)	0.73; 0.66
	Left eye	-0.08±0.12 -0.08 (0.11)	-0.08±0.14 -0.12 (0.14)	-0.13±0.10 -0.14 (0.21)	-0.10±0.07 -0.10 (0.10)	-0.10±0.10 -0.11 (0.12)	0.31; 0.60
	Both eyes	-0.16±0.09 -0.17 (0.09)	-0.15±0.09 -0.16 (0.14)	-0.21±0.07 -0.24 (0.11)	-0.16±0.08 -0.16 (0.12)	-0.16±0.08 -0.16 (0.14)	0.31; 0.32
**Near visual acuity^‡^** **(logMAR)**	Right eye	0.04±0.21 -0.02 (0.20)	-0.03±0.08 0.00 (0.10)	-0.05±0.05 -0.05 (0.10)	-0.05±0.06 -0.08 (0.10)	-0.03±0.11 -0.06 (0.10)	0.08; 0.28
	Left eye	0.01±0.11 0.00 (0.20)	-0.06±0.06 -0.10 (0.10)	-0.10±0.06 -0.10 (0.04)	-0.05±0.06 -0.10 (0.10)	-0.05±0.08 -0.10 (0.10)	0.42; 0.84
	Both eyes	-0.08±0.10 -0.10 (0.15)	-0.12±0.06 -0.10 (0.08)	-0.16±0.04 -0.17 (0.07)	-0.13±0.05 -0.10 (0.08)	-0.12±0.07 -0.10 (0.08)	0.07; 0.83
**Contrast sensitivity^‡^** **(log units)**	Right eye	1.91±0.11 1.95 (0.00)	1.95±0.02 1.95 (0.00)	1.94±0.02 1.95 (0.01)	1.93±0.07 1.95 (0.00)	1.93±0.06 1.95 (0.00)	0.42; 0.58
	Left eye	1.91±0.11 1.95 (0.00)	1.95±0.05 1.95 (0.05)	1.93±0.03 1.95 (0.05)	1.94±0.02 1.95 (0.00)	1.94±0.05 1.95 (0.00)	0.91; 0.73
	Both eyes	1.99±0.10 1.95 (0.11)	1.99±0.09 1.95 (0.10)	1.96±0.05 1.95 (0.04)	1.99±0.08 1.95 (0.00)	1.99±0.08 1.95 (0.04)	0.49; 0.66
**TNO**^**‡**^ **(sec of arc)**		135.0±155.5 60 (172.50)	75.0±60.4 60 (0.00)	110.0±70.1 90 (90.00)	123.9±117.5 60 (210.00)	111.8±107.960 (60)	0.22; 0.60
**Accommodation facility^‡^ (cpm)**		8.50±4.34 9.50 (9.25)	8.73±4.15 10.00 (4.00)	16.50±6.09 14.5 (13.00)	9.37±5.96 10.00 (4.00)	10.02±5.76 10.00 (4.00)	0.43; 0.27
**Vergence facility^‡^ (cpm)**		12.5±1.41 13.00 (2.50)	11.46±1.04 11.00 (1.00)	8.67±4.32 10.00 (8.00)	12.53±2.95 13.00 (2.00)	11.73±2.85 12.00 (2.80)	0.59; 0.07
**Color vision deficit^§^ (%)**		12.5	9.1	0	0	4.5	N/A

^†^Variable with normal distribution

^‡^Variable with non-normal distribution

^§^Categorial variable; N/A: not applicable. cpm: cycles per minute. Performance is represented by mean and standard deviation (upper numbers in each cell above) and by median and interquartile range [median (IQR), lower numbers in each cell above]. The interquartile range values are represented in parentheses. None of the p-values indicate statistically significant differences (i.e. all p-values were >0.004 (i.e. 0.05/12)) between AR and R, or between international and national-level officials.

### Relationship between clinically-measured and perceived levels of vision

Considering the data from all 71 participants QoV scores for *frequency* (r = -0.38, p = 0.001) and *severity* (r = -0.33, p = 0.005) were inversely correlated with PVVS (two tailed; with critical *p*-value of 0.008 (i.e. 0.05/6-pairwise-comparisons) after Bonferroni adjustment. This is sensible given that higher PVVS scores signify better perceived level of vision whereas lower QoV scores signify better perceived vision. However, the PVVS- QoV (*bothersome*) correlation did not reach statistical significance (r = -0.30, p = 0.012). QoV measures were significantly correlated with each other (r≥0.63, and all significant at the 0.001 level, two tailed).

Considering the data from the 44 subjects in whom both perceived vision scores and clinical data were available, PVVS scores were significantly correlated with distance VA in the left eye (r = -0.46, p = 0.002), but not in the right eye (r = -0.23, p = 0.129) or in both eyes (r = -0.35, p = 0.019) where comparisons were two-tailed and where the criterion *p*-value is 0.004 (i.e. 0.05/12-clinical-tests) after Bonferroni adjustment. PVVS scores were not significantly correlated with any other clinical vison measure and QoV scores did not correlate with any clinical vison measures (all r<0.3).

The ratings of officials by the Portuguese Federation did not correlate with QoV parameters (for R: frequency: r = 0.00, severity: r = -0.05, bothersome: r = -0.07, all p>0.77; for AR: frequency: r = -0.13, severity: r = -0.03, bothersome: r = -0.02, all p>0.39), or with PVVS (for R: r = -0.23 and AR: r = -0.05, both p>0.27) or with any clinical vision measure (for R and AR all p>0.12).

### Visual acuity using pinhole disc

With the pinhole disc, we identified eleven (11/44, 25%) individuals who showed an improvement of 5 or more letters (i.e. one line) improvement or more on the ETDRS chart in at least in one eye (Table 4). A one-line improvement represents the minimum improvement that can be considered significant [24, 25]; of these 11 participants, 4 wore refractive correction whilst officiating and 1 had not had an eye exam in the past three years and did not wear refractive correction whilst officiating. Table 4 also shows that twelve individuals (12/44, 27.3%) had an inter-eye VA difference of 5 or more letters on the ETDRS chart. However, with the pinhole disc, the inter-ocular difference was less than 5 letters in all of 12 cases. These results with the pinhole disc tentatively suggest that significantly better VA (i.e. ≥1 line improvement) may be achieved with a new or updated visual correction in about one-quarter of individuals of our sample. Across all participants, there was no correlation between inter-eye VA difference and stereoacuity (Distance VA vs TNO r = 0.28 and Near VA and TNO r = -0.09, both p>0.05).

**Table 4:** Results obtained with the pinhole disc.

	Referees		Assistant Referees		All n = 44
	International n = 8	National n = 11	International n = 6	National n = 19	
**Improvement of 5 or more ETDRS letters with the pinhole disc (%)**	3 (37.5)	2 (18.2)	1 (16.7)	5 (26.3)	11 (25)
**Absolute difference in distance VA between RE and LE of ≥ 1 line of ETDRS letters (%)**	4 (50.0)	3 (27.3)	2 (33.3)	3 (15.8)	12 (27.3)
**Numbers (%) who showed an improvement of 5 or more ETDRS letters with the pinhole disc AND who showed an absolute difference in distance VA between RE and LE of ≥ 1 line of ETDRS letters**	3 (37.5)	2 (18.2)	0 (0.0)	3 (15.8)	8 (18.2)

### Discussion

In Portugal, football players represent more than one quarter of all registered athletes [31]. In addition, football is one of the most important economic activities representing more than 0.15% of the Portuguese Gross National Product [32]. Officials are crucial participants in this activity. Characterizing and evaluating their vision is critical to ensure that vision is not a limiting factor in their performance on the field.

Two questionnaires to measure perceived level of vision (QoV, PVVS) and six clinical tests regularly used in eye examinations (visual acuity at distance and near, contrast sensitivity, stereoacuity, accommodation facility, vergence facility and colour vision) were administered to elite-level referees and assistant referees. Although the visual demands of acting as a referee and assistant referee are similar, they are not the same. In relation to both perceived level of vision and clinically-measured vision, no differences were apparent between referees and assistant referees. However, using the QoV, international-level officials may experience symptoms that are lower in severity compared to officials who operate exclusively in the national leagues.

### Frequency of eye examinations

Around one fifth (18.3%) of our participants had an eye examination more than three years ago, or had never had one. We know of no previous study that has examined the frequency with which elite-football officials present for eye examination. Interestingly, there is evidence that a sizeable proportion of high-level athletes also do not undergo regular eye checks. For example, Laby and colleagues found that in a large sample of US Olympic-level athletes, 48% had not had a recent eye examination and 25–33% had never had an examination [33]. Given the likely importance of good vision to participation in, and officiation of, sport at the highest level, it interesting to ask why such a sizeable proportion of high-level athletes and officials do not attend for eye examinations more regularly. In our sample, a failure to attend for regular eye examination by some elite may have resulted from the fact that they passed the ocular element of the annual CAME examination; however, this visual assessment goes little beyond VA assessment and includes no check of ocular health.

### Clinically-measured levels of vision

Twenty-eight percent of our participants had spectacles (spectacles only or both spectacles and contact lenses), which is less than the 41.3% reported for the Portuguese population aged 25–44 years [34]. Overall, 4.2% of our participants had undergone laser surgery and this figure is similar to the 5% found in the Portuguese population [35]. Around one quarter (23.9%) of the study population wear contact lenses, a figure that is about three times greater than in the Portuguese population of similar age (8%) [35]. This is likely to be related to the fact that when officiating, and when in need of refractive correction, football officials chose contact lenses over glasses, perhaps because vision in glasses is likely to be more affected by weather conditions (e.g. when raining). In all cases where a refractive correction was worn on the pitch, this consisted of contact lenses.

The near and distance VA, CS, stereoacuity, binocular accommodative facility, vergence facility and colour vision results obtained in this study were similar to results previously reported in young-adult populations (Table 5), suggesting that it is not necessary to exhibit elite performance in these standard, clinical visual measures to officiate in elite-level, Portuguese and European/world football. The fact that the elite-level officials in our study did not exhibit better than normal performance in the vision tests we employed is perhaps not surprising for a number of reasons. Firstly, standard clinical testing conditions are not replicated in match conditions where the viewing time is limited, the objects of interest are in motion and the environmental conditions (e.g. contrast and luminance) often differ markedly from those in clinical settings. Secondly, while the visual functions we measured are standard tests of ‘vision’, non-standard tests of vision may better reflect the demands of tasks required on the field. This is discussed further below (see *Limitations* section).

**Table 5:** Published normative test performative for young adults compared to performance averages exhibited by participants in the present study.

Test	Study	Subjects	Observations	Mean±SD* and 95% confidence interval in parantheses
**ETDRS** **Distance visual acuity** **(logMAR)**	Greene & Madden[36]	24 healthy subjects; 50% were female; mean age ± SD: 19.5 ± 1.9 years; wearing best correction	Binocular	-0.11±0.03 (-0.12 to -0.10)
	Coffey & Reichow[37]	650 Olympic athletes; 28% were females; Mean age ± SD: 21.1±5.5; wore their appropriate refractive correction	Monocular	-0.03±0.13 (-0.04 to -0.02)
			Binocular	-0.08±0.08 (-0.11 to -0.05)
	Elliott et al.[38]	13 healthy subjects; age range: 35–39 years; wearing optimal refractive correction	Monocular	-0.14±0.07 (-0.18 to -0.10)
	Ohlsson & Villarreal[39]	107 normal subjects; 66% were female; age range: 17–18 years; best corrected VA	Monocular	-0.12±0.07 (-0.13 to -0.11)
	Present Study	See *Methods*	Monocular (RE)	-0.10±0.08 (-0.12 to -0.07)
			Binocular	-0.16±0.08 (-0.19 to -0.14)
**ETDRS** **Near visual acuity** **(logMAR)**	Greene & Madden[36]		Binocular	-0.10±0.07 (-0.13 to -0.07)
	Elliott & Flanagan[22]	Considering the eye is accommodating normally or that the reading addition is correct	Similar to distance visual acuity	
	Present Study	See *Methods*	Monocular (RE)	-0.03±0.11 (-0.06 to 0.00)
			Binocular	-0.12±0.07 (-0.14 to -0.10)
**Pelli-Robson Contrast sensitivity** **(log units)**	Elliott et al.[40]	30 young subjects; mean age ± SD: 22.5 ± 4.3 years; full spectacles prescription	Monocular (dominant eye)	1.88 ± 0.08 (1.85 to 1.91)
	Elliott & Flanagan[22]		Binocular	If RE = LE then binocular = monocular +0.15
	Haymes et al.[41]	47 normal subjects; 53% were females; age range: 22–77 years; mean age: 48±17 years; test was performed with optimal spectacle refractive error correction	Monocular	1.79±0.11 (1.76 to 1.82)
	Beck et al.[42]	140 normal subjects; Mean age 32.3 ± 7.4 years; 74% women	Monocular	≥1.75
	Present Study	See *Methods*	Monocular (RE)	1.93±0.06 (1.91 to 1.95)
			Binocular	1.99±0.08 (1.96 to 2.01)
**Stereoacuity** **TNO** **(seconds of arc)**	Garnham & Sloper[43]	60 normal subjects; age range 17–83 years; wore their appropriate refractive correction		Median: 60
	Heron et al.[44]	51 normal subjects; age range 20–22 years		Median: 32
	Yekta et al.[45]	30 subjects; age range 30–39 years; wore their appropriate refractive correction		59.0±5.57
	Present study	See *Methods*		Median: 60 (60 to 120)
**Binocular accomodative facility:** **±2.00D flippers** **(cpm)**	Scheiman & Wick [46]	Normal adult subjects		10.0±5.0
	Ghasemi et al.[8, 9]	20 experienced football referees; mean age: 36.0±1.2 years.		12.0±3.9 (10.2 to 13.8)
	Zellers et al.[47]	100 normal adult subjects; corrected to 20/30 monocularly		7.7±5.2 (6.7 to 8.7)
	Present study	See *Methods*		10.0±5.8 (8.3 to 11.8)
**Vergence facility: 3**^**Δ**^ **base-in and 12**^**Δ**^ **base-out prism flippers**	Scheiman & Wick [46]	Normal adult subjects		15.0±3.0
	Gall et al.[48]	20 normal adult subjects; age range 18–35 years		16.0±2.6 (14.8 to 17.2)
	Present study	See *Methods*		11.7±2.9 (10.7 to 12.6)
**Colour vision deficiency (% of males exhibiting colour vision deficiency)**	Simunovic[49]	Meta-analysis	2% to 8%	
	Present study	See *Methods*	4.5% (0% to 10.5%)	

*Where no SD is listed, no SD was provided.

### Perceived level of vision

This is the first study to assess the subjective quality of vision in sports officials using the QoV and PVVS questionnaires. Compared to other questionnaires for visual function, assessment using the QoV has important advantages [15]. This questionnaire provides standardized photographs to simulate visual symptoms and this helps to promote better understanding and uniformity of concepts among respondents. In addition, it has the advantage that Rasch analysis was used in its development [12, 50, 51]. Unlike the QoV, the PVVS classifies vision globally in a single question [13].

Whereas the PVVS scores amongst our sample are in the last quartile of PVVS’s scale (i.e. implying a good level of perceived vision), our QoV scores, surprisingly, reflected a much lower perceived level of vision when compared with other samples of visually normal adults [52]. For example, in a recent study performed in Portugal of patients who had received multi-focal intra-ocular lens implants, the control group exhibited mean QoV scores for *frequency*, *severity* and *bothersome* of 24.5, 21.6 and 18.4, respectively [20]. Also, in their sample of patients presenting for refractive surgery, McAlinden reported mean pre-operative QoV scores for *frequency*, *severity* and *bothersome* of 6.8, 6.2 and 4.9, respectively [52]. These average values are markedly lower than the equivalent scores in our sample (Table 2). Indeed the QoV scores in our sample are even higher (implying poor perceived levels of vision) than in pre-operative patients with nuclear cataract where the mean QoV scores were 38.5, 33.1 and 35.9 for *frequency*, *severity* and *bothersome* [19]. This is intriguing since both referees and assistant referees had a good perception of their global vision, as evidenced by their PVVS scores, and overall good levels of vision as measured clinically (see above and Table 3). It is possible that the very high (i.e. poor) QoV scores could have resulted from questioning these individuals about visual symptoms which are specifically evaluated by the QoV and which are directly relevant to particular environmental conditions, which may frequently be sub-optimal, in which they operate (however, see limitations section below). Another possible explanation is that referees and assistant referees recognise the very high visual demands associated with their roles, and as a result have extremely high, perhaps unrealistic, expectations about the level of vision which they expect to achieve. However, this explanation seems unlikely as it probably would also have applied to the PVVS scores which show a much higher level of perceived vision compared to QoV scores.

The significant influence of subcategory (international or national) on one of the subscales of the QoV (*Severity*, Table 2) cannot be due to greater awareness or uptake of eye care since no difference existed in clinical vision measures and there was no greater frequency of eye exams amongst international level officials (Table 1).

Generically, our QoV scores were significantly correlated with PVVS scores (see results) and with each other. In relation to the latter, this might lead to speculation about a high level of redundant information in this type of data, an issue that was explored previously using the Bland-Altman limits of agreement method [53–55]. The three subscales of the QoV questionnaire were found to measure different aspects of the latent trait, as there were wide limits of agreement found between subscale scores suggesting that each subscale measures different aspects of the latent trait. The conclusion was that users should continue to use all three subscales of the questionnaire to achieve a comprehensive assessment of subjective quality of vision [55].

### Correlation between perceived vision and clinically-measured vision

There was little evidence for any correlation between measures of the perceived level of vision (PVVS and QoV) and clinical test measures. This suggests that the traditional clinical tests of vision used in a typical eye examination may not give an insight into the level of function as perceived by adults who are free from ocular disease and who have normal or close-to-normal vision. In individuals with cataract for example, there is a relationship between particular clinical measures self-reported visual difficulties [56, 57]. It is possible however, that the poor correlation between clinically measured vision and perceived- level of vision performance might be due to the fact that our study population had normal or close-to-normal levels of vision. Thus the absence of a meaningful relationship between perceived- and clinically-measured vision may be due to the narrow range of clinical levels of vision in our sample.

### Uncorrected and under-corrected refractive error

Using the pinhole, one quarter of participants in whom clinical visual measures were gathered showed an improvement in distance visual acuity of one line or more on the ETDRS chart (Table 4) which strongly suggests that, in these individuals, better VA may be achieved with appropriate (or more appropriate) visual correction. While some athletes may perform at a high level even with reduced VA [58], given the often large distance between the official and the play, sub-optimal VA could have a detrimental effect on the ability to gather the information needed to reach the correct decision. About 18% of the participants had an inter-eye difference in distance visual acuity of one line or more of the ETDRS chart, suggesting that these officials could benefit from a new or updated optical prescription for use on the pitch.

### Limitations of our study

The number of referees and assistant referees who completed the questionnaires represented a high proportion of the total number of elite-level, football officials in Portugal in the 2013/14 season (88% of Rs and 94% of ARs, and all international-level officials from both categories were included). However, in the time available to us, we were not able to gather clinical visual data for the entire sample, and this affected, in particular, the number of assistant referees for whom clinical data were available. This leads to two limitations. Firstly, the small size of some of the groups within our sample raises concerns about statistical power. Secondly, it is possible that the sample of assistant referees in whom we gathered clinical data was not representative of the entire assistant referee group. However, in relation to the latter, we feel the possibility of bias is mitigated by the pseudo-random manner in which we recruited assistant referees for clinical testing.

We assessed the potential to improve VA using the pinhole disc instead of conducting a full refraction, which would have told us directly about the prevalence and extent of uncorrected (and under-corrected) refractive error. The procedure we used allowed us to infer whether vision was optimal or could be improved but it does not accurately quantify the amount of possible improvement. However, the pinhole disc is an established method for assessing the scope for improved VA in the presence of uncorrected or under-corrected refractive error [23].

Our clinical data were similar to previously-reported, published results from young adult populations (Table 5) instead to a control group. However, we do not believe that this represents a significant limitation given that the reference values are well established in the literature and are widely known to the scientific and clinical communities.

A significant limitation of this study is that when the QoV and PVVS questionnaires were administrated, no specific instructions were given concerning whether the responses to be given should relate to vision when officiating as compared to in everyday life. The Portuguese version of the QoV that we used was translated [59], however this translated version has not, as yet, been independently validated. Our QoV results are sufficiently different from previously published values in adult populations that they warrant replication in a new population of football officials, in which they are asked specifically to complete the questionnaire based upon their perceived level vision whilst officiating.

The tests we included are standard clinical tests of vision. It has been argued that these tests are inadequate for the visual demands of elite athletes [60] and this may also be the case in elite sports officials. To assess the visual function of our sample of elite-level football officials, one could argue that we should have included tests that better reflect the demands of the tasks at hand. For example, since the demands of football officiating are at distance rather than at near, distance stereoacuity may have been an appropriate test to include in our test battery. Also, discrimination tasks under conditions of reduced contrast and limited presentation time may also prove useful [60]. Other tests, many not familiar to eye care professionals, may also have a place in the evaluation of football officials. Such tests, although vision-mediated, reflect perceptual-cognitive abilities [61] which might be linked to the task of high-level officiating. Another example is multiple object tracking which has been linked with better performance in elite athletes [62], (but see [63]) though not in football officials. Our intention here was to report on the basic visual abilities and visual history of high-level football officials. Future work should consider an extended range of tests, both clinical and non-clinical, with the aim of examining visual capabilities that are perhaps more closely related to visual requirements on the field of play and to try to establish the lab-based, perceptual-cognitive tasks which may underlie the tasks involved in football officiating.

Despite the limitations acknowledged above, we believe that our study makes a significant contribution to the knowledge of the visual performance of this population of elite-level, football officials as studies of this nature are rare in the scientific literature.

## Summary and conclusions

We studied the vision of elite-level football officials. The uptake of eye examination amongst this elite sample is variable. Around 1 in 5 officials are not having a regular exam. Around one-quarter wear refractive correction when officiating and in all cases this consisted of contact lenses. Around one-quarter appear to have visual function which may be improved through a new or updated refractive correction. On average, the vision of this sample is no better, but no worse than that which would be expected in similarly-aged adults. Although clinical vision measures are similar in referees and assistant referees, there was better perceived (QoV) vision in relation to severity of symptoms in international-level compared to national-level officials. Further studies are needed to understand the apparently poor level of perceived vision amongst football officials that we report using the QoV questionnaire and to understand how interventions to improve in clinical vision may alter officials’ perceived level of vision and performance on the field.

## Disclosure

The Quality of Vision questionnaire used in this study was developed by Colm McAlinden and colleagues in 2010 and Colm McAlinden was also co-author of this study. The authors report no conflicts of interest and have no proprietary interest in any of the materials mentioned in this article. This work was supported by the Portuguese Foundation for Science and Technology (FCT) in the framework of the Strategic Funding UID/FIS/04650/2013

## Supporting information

S1 Table Global vision status (PVVS) data and quality of vision questionnaire (QoV) data for each participant. (PDF)

S2 Table Clinical data for each participant. Same ID number in S1 and S2 indicates same participant. RE: right eye; LE: left eye; AF: accommodation facility; VF: vergence facility; cpm: cycles per minute.
(PDF)
