# Supplementary material for: Vision in high-level football officials
Source: PLoS One. 2017 Nov 21;12(11):e0188463. doi: 10.1371/journal.pone.0188463 (PMC5697857; doi:10.1371/journal.pone.0188463)
Supplement: S1 Table — (PDF) [file pone.0188463.s001.pdf]

ID	PVVS (0 to 10)	QoV		
		Frequency	Severity	Bothersome
1	9	64	57	71
2	9	32	27	38
3	8	45	44	53
4	9	32	13	14
5	8	67	57	65
6	9	45	39	42
7	8	72	52	78
8	9	32	13	0
9	6	56	57	71
10	9	61	52	57
11	9	59	54	63
12	9	61	66	78
13	8	52	39	34
14	8	64	78	96
15	8	59	78	93
16	8	69	70	83
17	9	59	42	46
18	8	69	54	63
19	5	75	57	65
20	8	52	42	46
21	7	69	68	75
22	9	52	42	46
23	6	72	68	73
24	8	49	39	60
25	7	56	49	50
26	8	52	44	50
27	8	52	54	63
28	8	64	47	53
29	8	64	61	85
30	9	25	22	23
31	8	61	44	50
32	9	67	47	53
33	8	59	52	73
34	7	64	52	65
35	7	72	54	65
36	8	52	42	50
37	8	77	57	65
38	7	64	32	23
39	8	59	54	63

40	9	64	49	57
41	10	56	42	46
42	8	61	59	73
43	3	59	44	50
44	7	77	75	88
45	8	59	63	65
46	9	87	63	75
47	9	52	70	83
48	8	67	66	71
49	8	72	72	85
50	10	72	59	65
51	10	75	66	75
52	9	37	27	29
53	8	59	59	65
54	7	72	61	65
55	9	59	42	57
56	8	59	70	85
57	9	61	52	65
58	10	25	22	29
59	8	59	63	73
60	7	69	63	75
61	8	64	47	34
62	7	64	70	80
63	8	45	42	42
64	9	61	54	71
65	9	41	32	29
66	8	49	32	29
67	9	41	35	34
68	9	52	75	96
69	7	64	57	65
70	10	32	22	14
71	9	64	52	60
