# Supplementary material for: Vision in high-level football officials
Source: PLoS One. 2017 Nov 21;12(11):e0188463. doi: 10.1371/journal.pone.0188463 (PMC5697857; doi:10.1371/journal.pone.0188463)
Supplement: S2 Table — Same ID number in S1 and S2 indicates same participant. RE: right eye; LE: left eye; AF: accommodation facility; VF: vergence facility; cpm: cycles per minute. (PDF) [file pone.0188463.s002.pdf]

ID	Category/ Subcategory	Distance visual acuity (logMAR)			Pinhole disc visual acuity (logMAR)		Near visual acuity (logMAR)			TNO	Colour vision (normal)	Contrast sensitivity (log units)			AF (cpm)	VF (cpm)
		RE	LE	Both eyes	RE	LE	RE	LE	Both eyes			RE	LE	Both eyes		
1	R/Inter	-0.16	-0.26	-0.30	-0.18	-0.26	-0.10	0.00	-0.10	30	Yes	1.95	1.95	1.95	3	12
2	R/Inter	-0.16	-0.16	-0.20	-0.18	-0.18	-0.10	-0.10	-0.14	30	Yes	1.95	1.95	2.10	13	13
3	R/Inter	-0.08	-0.14	-0.14	-0.12	-0.12	-0.14	-0.10	-0.16	60	Yes	1.95	1.95	2.20	8	11
5	R/Inter	0.04	0.14	0.00	0.00	-0.04	0.10	0.20	0.00	240	Yes	1.95	1.95	1.95	12	14
6	R/Inter	-0.04	-0.12	-0.10	0.02	-0.16	0.10	0.10	0.00	480	No	1.65	1.65	1.90	3	10
7	R/Inter	-0.06	-0.04	-0.18	-0.08	0.00	0.00	0.00	-0.10	60	Yes	1.95	1.95	1.95	13	14
8	R/Inter	-0.16	-0.04	-0.16	-0.16	-0.18	-0.04	-0.10	-0.20	60	Yes	1.95	1.95	1.95	11	13
9	R/Inter	-0.16	-0.04	-0.18	-0.12	-0.16	0.50	0.10	0.10	120	Yes	1.95	1.95	1.95	5	13
10	R/Nat	-0.20	-0.24	-0.26	-0.14	-0.14	0.00	-0.10	-0.10	60	Yes	1.95	1.95	2.10	10	11
11	R/Nat	-0.06	-0.12	-0.16	-0.10	-0.16	-0.10	-0.10	-0.10	30	Yes	1.95	1.90	2.05	12	13
12	R/Nat	-0.16	-0.16	-0.16	-0.14	-0.14	0.00	-0.10	-0.14	60	Yes	1.95	1.95	2.00	8	12
13	R/Nat	-0.10	-0.08	-0.10	-0.08	-0.08	-0.10	-0.10	-0.10	60	Yes	1.95	2.10	1.95	2	10
14	R/Nat	-0.06	-0.18	-0.24	-0.14	-0.10	-0.10	-0.16	-0.18	60	Yes	1.95	1.95	1.95	0	11
15	R/Nat	-0.10	-0.10	-0.20	-0.10	-0.10	0.10	0.00	-0.10	15	Yes	1.95	1.95	1.90	9	12
16	R/Nat	0.10	0.22	0.04	0.00	0.04	0.10	0.00	0.00	120	Yes	1.95	1.90	1.90	12	11
17	R/Nat	-0.14	-0.12	-0.16	-0.16	-0.16	-0.10	-0.10	-0.10	60	Yes	1.95	1.95	1.95	11	10
18	R/Nat	-0.10	-0.04	-0.14	-0.14	-0.08	0.00	0.00	-0.12	240	Yes	1.90	1.90	2.20	12	12
19	R/Nat	-0.06	0.14	-0.06	-0.06	-0.04	-0.10	0.00	-0.18	60	No	1.95	1.95	1.95	8	11
22	R/Nat	-0.16	-0.20	-0.24	-0.18	-0.22	0.00	0.00	-0.20	60	Yes	1.95	1.95	1.95	12	13
24	AR/Inter	-0.04	0.00	-0.08	-0.08	0.04	0.00	-0.10	-0.20	120	Yes	1.95	1.90	1.90	24	2
25	AR/Inter	-0.16	-0.14	-0.24	-0.16	-0.14	-0.10	-0.10	-0.20	60	Yes	1.95	1.95	2.05	11	13
26	AR/Inter	-0.24	-0.14	-0.28	-0.16	-0.16	-0.10	-0.10	-0.10	60	Yes	1.95	1.95	1.95	16	5
29	AR/Inter	0.00	-0.04	-0.16	-0.10	-0.10	-0.10	-0.18	-0.18	240	Yes	1.90	1.90	1.95	13	9
30	AR/Inter	-0.10	-0.24	-0.24	-0.18	-0.16	0.00	0.00	-0.16	60	Yes	1.95	1.95	1.95	24	11

32	AR/Inter	-0.16	-0.24	-0.24	-0.16	-0.18	0.00	-0.10	-0.14	120	Yes	1.95	1.95	1.95	11	12
34	AR/Nat	0.12	-0.10	-0.10	-0.02	-0.10	-0.10	-0.10	-0.10	240	Yes	1.65	1.90	1.95	8	12
53	AR/Nat	-0.14	-0.06	-0.14	-0.12	-0.10	0.00	0.00	0.00	240	Yes	1.95	1.95	1.95	9	12
55	AR/Nat	-0.10	-0.16	-0.24	-0.12	-0.16	0.00	-0.10	-0.10	60	Yes	1.95	1.95	2.10	12	13
56	AR/Nat	-0.08	-0.06	-0.12	0.00	0.00	0.00	0.00	-0.10	120	Yes	1.95	1.95	1.95	13	13
57	AR/Nat	-0.14	-0.10	-0.14	-0.10	-0.10	-0.06	-0.10	-0.10	480	Yes	1.90	1.90	1.95	10	12
58	AR/Nat	-0.06	-0.16	-0.26	-0.16	-0.16	0.10	0.00	-0.10	120	Yes	1.95	1.95	1.95	0	2
59	AR/Nat	-0.14	-0.20	-0.30	-0.14	-0.18	-0.10	-0.10	-0.18	60	Yes	1.95	1.95	2.10	5	12
60	AR/Nat	0.00	0.02	-0.06	-0.06	-0.08	0.00	-0.10	-0.14	60	Yes	1.95	1.95	1.95	12	14
61	AR/Nat	0.02	-0.06	-0.06	-0.10	-0.10	-0.10	-0.10	-0.20	240	Yes	1.95	1.95	2.20	10	10
62	AR/Nat	-0.18	-0.16	-0.16	-0.08	-0.08	-0.10	-0.10	-0.18	30	Yes	1.95	1.95	1.95	0	14
63	AR/Nat	-0.26	-0.06	-0.26	-0.22	-0.16	-0.10	-0.10	-0.16	15	Yes	1.95	1.95	1.95	10	10
64	AR/Nat	-0.16	-0.16	-0.16	-0.14	-0.16	-0.12	-0.10	-0.14	60	Yes	1.95	1.95	1.95	9	14
65	AR/Nat	-0.06	0.02	-0.06	-0.06	-0.04	-0.06	-0.08	-0.10	120	Yes	1.95	1.95	1.95	27	15
66	AR/Nat	-0.14	-0.16	-0.16	-0.10	-0.10	-0.10	0.00	-0.10	30	Yes	1.95	1.95	1.95	12	15
67	AR/Nat	-0.18	-0.14	-0.20	-0.16	-0.16	0.00	0.00	-0.10	60	Yes	1.90	1.90	1.95	14	15
68	AR/Nat	-0.04	-0.10	-0.10	-0.08	-0.14	0.00	0.00	-0.10	120	Yes	1.95	1.95	1.95	8	14
69	AR/Nat	0.00	0.00	-0.06	0.00	0.00	-0.10	-0.10	-0.20	240	Yes	1.95	1.95	1.95	10	14
70	AR/Nat	-0.06	-0.06	-0.20	-0.06	-0.08	-0.10	0.00	-0.20	30	Yes	1.95	1.95	2.10	1	14
71	AR/Nat	-0.20	-0.20	-0.22	0.00	0.00	-0.08	0.10	-0.10	30	Yes	1.95	1.95	1.95	8	13
